# Self-Refining Segment Anything Model for Nuclei Segmentation as Contrastive Learning Approach to Label-Efficient Pathological Imaging

**DOI:** 10.3390/diagnostics16091370

**Published:** 2026-04-30

**Authors:** Siwoo Nam, Sang Hyun Park

**Affiliations:** 1 Department of Robotics and Mechatronics Engineering, Daegu Gyeongbuk Institute of Science and Technology (DGIST), Daegu 42988, Republic of Korea; siwoonam@dgist.ac.kr; 2 Department of Computer Science and Engineering, Pohang University of Science and Technology (POSTECH), Pohang 37673, Republic of Korea

**Keywords:** nuclei instance segmentation, Segment Anything Model (SAM), weakly supervised learning, contrastive learning, pseudo-labeling

## Abstract

**Background/Objectives**: Precise nuclei instance segmentation is a prerequisite for reliable digital pathology, yet the scarcity of pixel-level annotations remains a significant bottleneck for deep learning models. **Methods**: We propose a self-evolving framework for robust nuclei segmentation that uses only sparse point annotations, extending the Segment Anything Model (SAM). To overcome the limitations of static pseudo-labels, our method introduces a self-evolving labeling strategy via Exponential Moving Average (EMA), which adaptively refines learning targets. We also integrate instance-aware contrastive learning using point prompts as spatial anchors and implement a consensus-based filtering mechanism between prompt-guided and prompt-free decoders. **Results**: Extensive evaluations on CPM17, MoNuSeg, and the challenging CoNSeP datasets demonstrate that our framework achieves state-of-the-art performance across various backbones, including ViT-B and ViT-H. **Conclusions**: By enabling a seamless transition from general-purpose foundation models to specialized histopathology experts, this self-refining approach delivers a highly efficient, accurate solution for automated diagnostic workflows in clinical settings.

## 1. Introduction

Computer-aided analysis of histopathology images is essential for modern cancer diagnosis, including tasks such as tumor grading and treatment planning [1,2,3]. Recently, advanced deep learning models have been developed for specialized diagnostic tasks, such as laryngeal tumor grading using deformable fusion attention (DCA-DAFFNet) [4], knowledge-driven multiple instance learning [5], or hierarchical model fusion (ViT-AMC, MamlFormer) [6,7]. However, these high-level diagnostic systems depend heavily on the quality of basic feature extraction. Specifically, precise nuclei instance segmentation is the most important foundation for these systems; if individual cells are not accurately segmented, subsequent diagnostic analysis and grading results become unreliable.

To achieve accurate segmentation, many researchers have utilized fully supervised learning [8,9,10,11,12,13,14]. Although effective, these methods require thousands of pixel-level manual annotations, which is a slow and labor-intensive process. To reduce this burden, weakly supervised learning (WSL) using simple point annotations has become a popular alternative [15,16,17,18]. Early WSL methods often relied on simple geometric rules, such as Voronoi diagrams or K-means clustering [19], but they frequently fail to separate clustered nuclei or handle the complex cell shapes found in cancerous tissues.

The Segment Anything Model (SAM) has recently emerged as a powerful tool for general image segmentation [20]. However, SAM often performs poorly in digital pathology because of the large domain gap between natural images and microscopic tissue slides [21,22,23,24]. To adapt SAM for medical use, parameter-efficient fine-tuning (PEFT) strategies like InstaSAM [25] have been proposed. Although InstaSAM can learn from point prompts, it uses static pseudo-labels generated only at the very start of training. This means that the model cannot improve its own targets as it learns, often resulting in a performance limit due to the initial errors of the frozen SAM model.

In this study, we propose a novel self-evolving framework that transcends the limitations of static pseudo-labeling in weakly supervised nuclei segmentation. Our method introduces a Self-evolving Pseudo-labeling strategy using Exponential Moving Average (EMA), allowing the model to adaptively refine its target labels by integrating specialized knowledge acquired during training. By evolving from generic zero-shot outputs to domain-specific refined labels, our model achieves superior stability and accuracy. Furthermore, we incorporate instance-aware contrastive learning by utilizing point prompts as stable spatial anchors. To avoid assigning a pseudo-label to all ignored regions, our contrastive module provides minimal guidance in the latent space. This representational refinement is crucial for maintaining training stability and resolving atypical shapes where the SAM often fails to generate valid masks. Moreover, to optimize training efficiency, we present a hierarchical pseudo-label refinement mechanism. By evaluating the agreement between the prompt-guided mask decoder and a prompt-free nuclei decoder, we strategically reduce the ignored regions that typically hinder the convergence of weakly supervised models. This consensus-driven approach maximizes data utilization and significantly accelerates training speed. We validate the robustness of our framework across multiple benchmarks, including CPM17 [26], MoNuSeg [27], and CoNSeP [9], demonstrating state-of-the-art performance across various backbones.

The main contributions of this work are as follows:

- We propose a self-evolving pseudo-labeling strategy using EMA, enabling the model to refine its own learning targets and overcome the noise inherent in initial zero-shot labels.
- We introduce an instance-aware contrastive learning module that utilizes point prompts as reliable spatial anchors, providing essential latent-space supervision even when foreground pseudo-labels are missing or filtered out.
- We design a hierarchical pseudo-label refinement mechanism that differentiates between prompt-based adaptation and prompt-free segmentation targets to recover reliable pixels from previously ignored regions, significantly enhancing training efficiency and data utilization.
- We demonstrate the superior generalizability and robustness of our framework through extensive experiments on multiple histological datasets and various Vision Transformer backbones.

## 2. Materials and Methods

### 2.1. Overall Architecture

**Figure 1:**
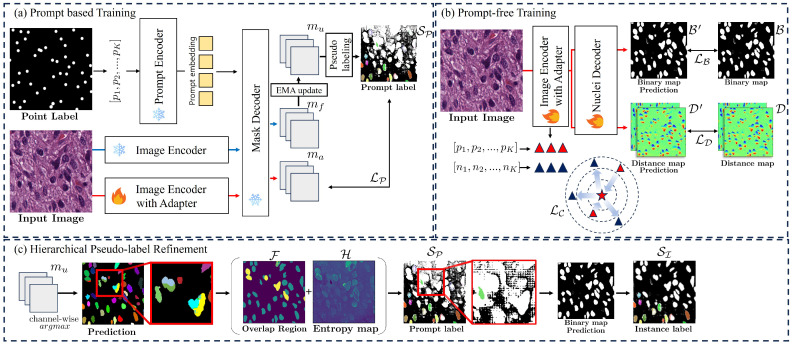
Overview of the self-evolving SAM-based framework. (**a**) Prompt-based training fine-tunes adapters in the frozen SAM encoder using EMA-refined mask predictions. (**b**) Segmentation training optimize nuclei decoder for prompt-free instance segmentation, incorporating instance-aware contrastive learning. (**c**) Pseudo-labeling process comprises consensus-driven filtering that refines pseudo-labels at both stages. In the architecture overview, the flame icon denotes trainable modules, while the snowflake symbol indicates frozen components. Triangles represent features extracted from the image encoder with adapters, and the star indicates the learnable shared prototype. B and D are the binary and distance pseudo-ground-truth maps generated from the instance label $S_{I}$.

As illustrated in Figure 1, the proposed framework is designed to achieve robust nuclei instance segmentation under weak supervision by integrating three core components: prompt-based domain adaptation, prompt-free instance segmentation, and hierarchical pseudo-label refinement. To establish a formal logical flow, we define the following notations for the primary modules: let $E_{base}$ be the frozen SAM image encoder, $E_{adapt}$ be the image encoder equipped with trainable adapter layers, $\Phi_{mask}$ be the SAM mask decoder for prompt-guided tasks, and $\Phi_{nuclei}$ be the trainable nuclei decoder for prompt-free prediction.

The algorithmic flow of our framework operates through the organic interaction between these components. First, given an input image *I*, we extract two sets of latent representations: domain-agnostic embeddings $z_{base}=E_{base}\left(I\right)$ and domain-specific embeddings $z_{adapt}=E_{adapt}\left(I\right)$. In the prompt-based adaptation stage shown in Figure 1a, $\Phi_{mask}$ utilizes sparse point prompts *P*, $z_{base}$ and $z_{adapt}$ to generate initial masks $m_{u}$, which are then refined through an Exponential Moving Average mechanism to produce stable learning targets, as detailed in Section 2.2. Simultaneously, the hierarchical refinement process in Figure 1c evaluates the consensus between the outputs of $\Phi_{mask}$ and $\Phi_{nuclei}$ to minimize ignored regions and recover reliable training pixels, which is further elucidated in Section 2.4. These refined labels serve as supervision for the segmentation training stage in Figure 1b, where $\Phi_{nuclei}$ is optimized using $z_{adapt}$ alongside an instance-aware contrastive learning objective to ensure feature discriminability, as described in Section 2.3. Comprehensive implementation details, including dataset configurations and the experimental environment, are provided in Section 2.5.

### 2.2. Prompt-Based Domain Adaptation

The first stage of our framework, as illustrated in Figure 1a, aims to bridge the domain gap between natural images and histopathology slides by leveraging the zero-shot capabilities of SAM through point-guided adaptation. In this process, the input image *I* is fed into $E_{adapt}$, which is augmented with lightweight, trainable adapter layers [28] to extract domain-specific image embeddings $z_{adapt}$ while the original backbone parameters remain frozen. Simultaneously, the sparse point annotations $P=\{p_{1},p_{2},\ldots ,p_{K}\}$ are processed by the frozen prompt encoder to generate corresponding prompt embeddings. These embeddings are then integrated within $\Phi_{mask}$ to produce the adapted mask predictions $m_{adapt}$.

The primary objective of this phase is to refine the image embeddings to capture nuclei-specific morphological features. However, a significant challenge in weakly supervised nuclei segmentation is the inherent noise in the zero-shot labels generated by the frozen SAM model. Unlike previous approaches that rely on a static target [25], we propose a self-evolving labeling strategy using an Exponential Moving Average (EMA) mechanism. Let $m_{base}=\Phi_{mask}\left(z_{base},P\right)$ be the initial prediction derived from the frozen SAM image encoder. To ensure stable convergence while allowing the model to escape the constraints of the initial zero-shot output, we define a refined instance prediction $m_{u}$ as:

(1) $$m_{u}=\alpha_{t}m_{base}+\left(1-\alpha_{t}\right)m_{adapt}$$

we define $\alpha_{t}$ as a linearly decaying coefficient: $\alpha_{t}=\max\left(0,1-t/T\right)$, where *t* and *T* denote the current and total epochs, respectively. This scheduling ensures that the model gradually shifts its reliance from the initial frozen SAM outputs to its own self-adapted predictions. From these refined predictions $m_{u}$, we generate the prompt label $S_{P}$ by applying the consensus logic detailed in Section 2.4. The prompt-based loss $L_{P}$ is defined as the discrepancy between the adapted output $m_{adapt}$ and the refined label $S_{P}$. The loss for the *k*-th instance is computed as:

(2) $$L_{P}=\frac{1}{K}\sum_{k=1}^{K}\left(L_{BCE}\left(m_{adapt,k},S_{p,k}\right)+L_{IoU}\left(m_{adapt,k},S_{p,k}\right)\right)$$

where $m_{adapt,k}$ is the predicted mask for the *k*-th nucleus. By minimizing $L_{P}$, the adapter layers learn domain-specific representations that align with the high-confidence regions of the evolving pseudo-labels. This self-correction loop ensures that $m_{adapt}$ becomes increasingly reliable, serving as the critical source for generating the Instance Label $S_{I}$ used in the subsequent prompt-free segmentation phase. Even in cases where $m_{base}$ provides poor initial guidance, the synergy between this evolving label and contrastive learning anchors (detailed in Section 2.3) provides a minimum safeguard to maintain representational stability.

### 2.3. Prompt-Free Instance Segmentation

The second stage of our framework, as depicted in Figure 1b, focuses on training the nuclei decoder $\Phi_{nuclei}$ to perform instance segmentation during inference without requiring point prompts. While $\Phi_{mask}$ focuses on individual instances via prompts, $\Phi_{nuclei}$ learns to capture the global distribution and geometric properties of all nuclei within the input image. This decoder utilizes a dual-head architecture to predict: (1) a binary foreground map $B^{\prime }\in {\left[0,1\right]}^{H\times W}$ to identify nuclear regions, and (2) a distance map $D^{\prime }\in R^{2\times H\times W}$ for boundary delineation. $B^{\prime }$ is optimized using $L_{B}$ (BCE and IoU losses), while $D^{\prime }$ is trained via L1 regression $L_{D}$ to represent relative pixel distances from nuclear centers.

To enhance feature discriminability, we introduce an instance-aware contrastive learning objective. We employ a projection head $g_{\omega }\left(\cdot \right)$, consisting of a 2-layer MLP with ReLU activation, to map the adapted embeddings $z_{adapt}$ into a compact latent space $R^{d}$. For each nucleus *k*, we define a positive anchor $q_{k}$ as the projected feature at the coordinate of point prompt $p_{k}$. This anchor is pulled toward a single learnable prototype, $z^{+}\in R^{d}$, shared across all nuclei and updated via backpropagation to capture a representative morphological signature of the nuclei. Crucially, to address the ambiguity in weakly supervised labels, we employ hard-negative mining. Negative samples ${\left\{q_{j}^{-}\right\}}_{j=1}^{N}$ are extracted from hard background regions where the model mistakenly predicts high foreground probability ($B^{\prime }>0.7$) despite being labeled as background in the pseudo-labels. By randomly sampling up to 100 such hard negative pixels per batch, the contrastive loss $L_{C}$ is formulated as:

(3) $$L_{C}=-\frac{1}{K}\sum_{k=1}^{K}\log\frac{\exp(sim\left(q_{k},z^{+}\right)/\tau )}{\exp\left(sim\left(q_{k},z^{+}\right)/\tau \right)+\sum_{j=1}^{N}\exp\left(sim\left(q_{k},q_{j}^{-}\right)/\tau \right)}$$

where sim(·) denotes cosine similarity and τ=0.1 is the temperature scaling factor. This representational learning ensures that features remain distinct even in dense clusters, providing minimal guidance that prevents the model from collapsing when pseudo-masks are missing. The final multi-task objective is:

(4) $$L_{total}=\lambda_{P}L_{P}+\lambda_{B}L_{B}+\lambda_{D}L_{D}+\lambda_{C}L_{C}$$

### 2.4. Hierarchical Pseudo-Label Refinement

The efficacy of our weakly supervised framework stems from a hierarchical refinement process that generates stage-specific pseudo-labels. As shown in Figure 1c, we define two distinct sets of targets: the Prompt Label $S_{P}$ for domain adaptation and the Instance Label $S_{I}$ for training the nuclei decoder. This differentiation allows the model to first stabilize via sparse cues and subsequently expand its knowledge through cross-branch consensus.

During the prompt-based domain adaptation phase, we generate initial pseudo-labels $S_{P}$ to supervise the trainable adapters $E_{adapt}$. Following the conservative filtering strategy of previous work [25], we define an ignore mask $M_{p}$ to exclude ambiguous pixels. A pixel *j* is marked as ignored ($M_{p}\left(j\right)=1$) if it exhibits high uncertainty or spatial ambiguity:

(5) $$M_{p}\left(j\right)=1\;\mathrm{if}\;H\left(j\right)>0.3\;\mathrm{or}\;F\left(j\right)\geq 2$$

where $H\left(j\right)=-\sum m_{u}\cdot \log\left(m_{u}\right)$ represents the entropy of the EMA-refined prediction $m_{u}$, and F(j) denotes the number of overlapping instance masks. This strict filtering ensures that the adapter layers are trained only on high-confidence, non-overlapping nuclear regions, preventing the propagation of zero-shot errors from the frozen SAM image encoder.

For the prompt-free segmentation phase, we synthesize more comprehensive instance labels $S_{I}$ by evaluating the consensus between the prompt-guided output $m_{u}$ and the prompt-free prediction $B^{\prime }$. Unlike the static $M_{p}$, the ignore mask $M_{I}$ is designed to recover valid training pixels by leveraging the global semantic awareness of the nuclei decoder. We define foreground agreement $A_{fg}$ and background agreement $A_{bg}$ as sets where both branches reach a high-confidence consensus:

(6) $$\begin{array}{cc} A_{fg} & =\{j|\max\left(m_{u}\left(j\right)\right)>0.5\wedge B^{\prime }\left(j\right)>0.7\wedge F\left(j\right)<2\}\\ A_{bg} & =\{j|\max\left(m_{u}\left(j\right)\right)<0.2\wedge B^{\prime }\left(j\right)<0.3\} \end{array}$$

where ∧ denotes the logical AND operator. Following this, the final ignore mask $M_{I}$ is formally defined as:

(7) $$M_{I}=\left(I_{base}\cup F\right)\setminus \left(A_{fg}\cup A_{bg}\right)$$

where $I_{base}$ represents the set of pixels with high uncertainty or branch conflict:

(8) $$I_{base}=\left\{j|H\left(j\right)>0.3\vee \left(I\left(m_{u}\left(j\right)>0.5\right)\neq I\left(B^{\prime }\left(j\right)>0.5\right)\right)\right\}$$

Here, ∨ denotes the logical OR operator and I(·) is the indicator function. The thresholds for agreement (0.5, 0.7, 0.2, 0.3) were empirically selected to prioritize high-precision foreground and high-confidence background, ensuring that only the most reliable pixels are utilized for the expansion of the training signal. This hierarchical interaction allows the model to recover valid pixels that were initially ignored by $M_{p}$, providing $\Phi_{nuclei}$ with a denser and more accurate training signal, which is critical for robust prompt-free inference.

### 2.5. Datasets and Implementation Details

To evaluate the performance and generalizability of our framework, we utilized three independent histopathology datasets: CPM17, MoNuSeg, and CoNSeP.

- CPM17 [26]: This dataset consists of 64 H&E stained images from the 2017 Computational Precision Medicine challenge. It contains 7570 annotated nuclear boundaries with image sizes between 500×500 and 700×700 pixels. We utilized the standard split of 32 images for training and 32 images for testing.
- MoNuSeg [27]: This multi-organ dataset includes 30 H&E images (1000×1000 pixels) from 7 human organs. It contains 21,623 annotated nuclei. We followed the standard protocol by splitting the dataset into 16 training and 14 testing images.
- CoNSeP [9]: This dataset consists of 41 colorectal adenocarcinoma images of 1000×1000 pixels. Following the original protocol, we used 27 images for training and 14 for testing. This dataset is particularly challenging due to dense cell clusters and diverse nuclear morphologies.

To quantitatively assess segmentation performance, we employed two primary metrics: the Dice coefficient and the Aggregated Jaccard Index (AJI). The Dice coefficient measures the pixel-level overlap between the predicted mask and the ground truth. However, as pathology analysis requires precise separation of adjacent nuclei, we prioritize the AJI, which is specifically designed for instance segmentation. The AJI aggregates the intersection areas over all ground truth and predicted instances, effectively penalizing both false positives and merged instances.

The primary experiments were conducted using SAM with a ViT-H image encoder, while ViT-B and ViT-L backbones were utilized for ablation studies. All models were trained for 100 epochs with a batch size of 1 using the AdamW optimizer [29] and an initial learning rate of $1\times 10^{-4}$, which followed a CosineAnnealingLR schedule [30] with $t_{max}=20$. Regarding the self-evolving strategy, the momentum coefficient $\alpha_{t}$ was linearly decayed from 1.0 to 0 over 100 epochs, facilitating a gradual transition from the frozen SAM outputs $m_{base}$ to the self-adapted predictions $m_{adapt}$. For contrastive learning, the temperature τ was set to 0.1. For the multi-task loss optimization, the weights were empirically set as $\lambda_{P}=1,\;\lambda_{B}=1,\;\lambda_{D}=5$, and $\lambda_{C}=1$. To prevent overfitting and enhance robustness, we employed extensive data enhancement strategies, including random resizing, affine transformations (rotation, scaling), horizontal flipping, and random cropping. All experiments were implemented in PyTorch 1.13.1 and executed on a single NVIDIA RTX A6000 GPU.

## 3. Results

### 3.1. Quantitative Evaluation on Public Datasets

To evaluate the efficacy of our self-evolving framework, we conducted extensive experiments on three public histopathology datasets: CPM17, MoNuSeg, and CoNSeP. Table 1 summarizes the comparative results using the Dice coefficient and the Aggregated Jaccard Index (AJI) as primary metrics. To assess the robustness of the models against annotation noise, we report performance under both precise point annotations (Shift 0) and noisy annotations (Shift 8). As shown in Table 1, the proposed method consistently outperforms existing weakly supervised approaches across most benchmarks, especially in datasets characterized by high morphological diversity. On the CPM17 dataset, while our framework maintains competitive results, it achieves a slightly lower Dice score of 82.9% compared to InstaSAM of 83.9% at Shift 0. We attribute this marginal difference to the specific characteristics of the CPM17 dataset, where nuclei exhibit relatively regular and uniform shapes that align well with SAM’s initial zero-shot capabilities. In such near-optimal scenarios, the aggressive consensus filtering and EMA-based refinement—designed to bridge significant domain gaps—may introduce minor fluctuations in pseudo-labels. However, the true advantage of our self-evolving mechanism is clearly demonstrated in more complex and realistic pathological environments. Unlike CPM17, which is relatively homogeneous, the MoNuSeg and CoNSeP datasets encompass a wide variety of human organs and malignant cell classes with highly atypical morphologies. On the CoNSeP dataset, our model achieved an AJI of 45.3% at Shift 0, significantly outperforming InstaSAM (40.5%) and other point-supervised methods. Furthermore, in the MoNuSeg Shift 8 setting, our method maintained a robust AJI of 54.1%, effectively mitigating the performance degradation caused by annotation noise. These results prove that our framework excels at generalizing across diverse tissue types and organ domains where frozen foundation models typically fail, delivering a more reliable solution for complex automated diagnostic workflows.

### 3.2. Qualitative Analysis

**Figure 2:**
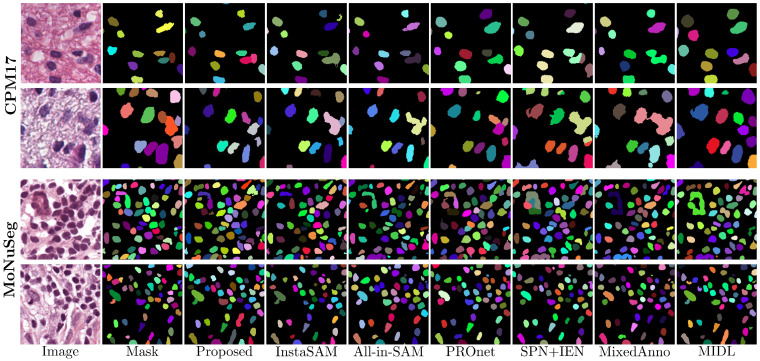
Qualitative comparison of CPM17 and MoNuSeg datasets. In the segmentation results, individual colors are assigned to different nuclei to represent distinct instances, facilitating the visualization of the model’s ability to separate clustered cells. ’Mask’ denotes the ground truth, while other columns represent predicted outputs from various models.

**Figure 3:**
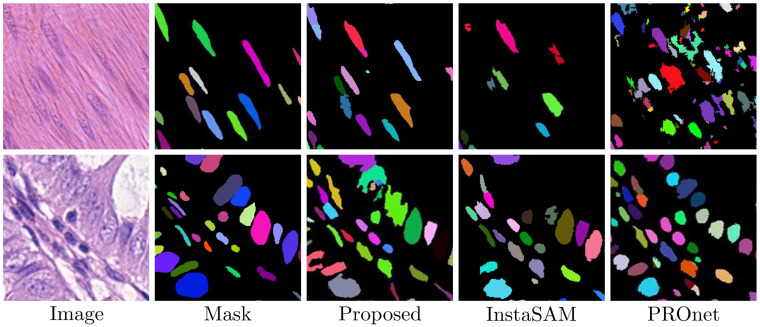
Qualitative results on the challenging CoNSeP dataset. Each color in the predicted masks denotes a unique nucleus instance, highlighting the morphological fidelity and instance-level accuracy of the proposed method in complex tissue structures.

Figure 2 and Figure 3 present a visual comparison of the segmentation results between the proposed method and baseline models across three datasets. The qualitative results highlight several key advantages of our framework regarding morphological fidelity and instance separation. As illustrated in Figure 2, the proposed model produces significantly smoother, more biologically plausible nuclear boundaries than previous methods. While InstaSAM often struggles to delineate individual nuclei in dense clusters, our model effectively separates spatially adjacent instances through more accurate distance map regression. Notably, our framework successfully recovers multiple instances that were entirely missed by InstaSAM. This improvement is primarily due to the instance-aware contrastive learning module, which provides minimum learning guidance in regions where pseudo-labels are initially masked as ignored. By utilizing point prompts as stable spatial anchors, the model maintains a fundamental representation of the nuclei even when explicit binary supervision is absent or filtered out. The superiority of our self-evolving mechanism is further demonstrated in the CoNSeP dataset at Figure 3, which features highly challenging nuclear shapes. For elongated nuclei, which are often indistinguishable even to the naked eye, zero-shot SAM backbones typically fail to generate valid masks. InstaSAM frequently fails to detect these instances, and PROnet often forces them into simplified circular masks, thereby failing to preserve their true biological morphology. In contrast, the proposed method accurately captures these atypical shapes by leveraging the synergy between EMA-based pseudo-label refinement and contrastive anchors. The EMA update rule allows the model to progressively refine its learning targets into domain-specific labels, while the contrastive loss ensures that the features for these challenging instances remain discriminative in the latent space. This combined approach ensures stable pseudo-label generation, allowing the model to overcome the inherent limitations of foundation models in specialized pathological imaging.

### 3.3. Ablation Study

To investigate the contribution of each proposed component, we conducted a step-by-step ablation study on the CPM17 and MoNuSeg datasets using a ViT-B backbone, comparing our framework against the InstaSAM baseline. As detailed in Table 2, each strategy yielded incremental gains in segmentation accuracy. The baseline InstaSAM model achieved an AJI of 63.49% on the CPM17 dataset. By introducing the EMA-based self-evolving labeling mechanism and the consensus-based filtering strategy—which together adaptively refine targets and recover reliable training pixels through cross-branch agreement—the performance improved to 64.18% AJI. The subsequent inclusion of the instance-aware contrastive loss further enhanced the results to a Dice score of 80.38% and an AJI of 65.15% on CPM17. These results confirm that the synergy between adaptive labeling via EMA and representational learning is crucial for capturing the complex morphological features of nuclei in pathological images.

We further evaluated the sensitivity of the momentum coefficient ($\alpha_{t}$) to various scheduling strategies to justify our choice of linear reduction. As shown in Table 3, while the Step-wise strategy performed competitively on the simpler CPM17 dataset, our proposed Linear decay strategy proved more robust on the more challenging MoNuSeg and CoNSeP datasets, achieving AJIs of 53.53% and 37.70%, respectively. This linear schedule addresses convergence stability by utilizing the frozen SAM output as a stable spatial prior during the initial stages of training. As the adapter layers specialize in the pathology domain, the gradual reduction of $\alpha_{t}$ facilitates a smooth transition to self-adapted predictions, preventing the model from being trapped in noisy local minima and ensuring a stable curriculum.

Finally, we assessed the scalability and computational efficiency of our framework across different Vision Transformer backbones on the CoNSeP dataset. As shown in Table 4, the proposed method consistently improved performance over the baseline regardless of the backbone size, with ViT-B even approaching the performance of larger baseline models. Regarding the computational overhead, our self-refining process is highly efficient. For the ViT-B backbone, the peak VRAM usage during training increased only marginally from 77.12 MB to 89.56 MB, representing a negligible overhead of approximately 12.4 MB. Furthermore, since our framework utilizes the same architecture as the baseline during the inference phase, it requires no additional computational cost or memory during deployment. These findings demonstrate that our approach is fully feasible on consumer-grade hardware, making it a practical solution for clinical environments with limited resources.

## 4. Discussion

The results of this study demonstrate that the proposed self-evolving SAM-based framework significantly enhances the accuracy and robustness of nuclei instance segmentation under weak supervision. By moving beyond the static pseudo-labeling constraints of earlier models [25], our approach effectively bridges the domain gap between general foundation models and specialized histopathological analysis.

The primary innovation of our work lies in the self-evolving pseudo-labeling strategy via EMA. Traditional weakly supervised methods, such as those relying on Voronoi diagrams or K-means clustering [15,19], are often limited by rigid geometric priors that do not account for the irregular morphologies of cancerous nuclei. While recent SAM-based adaptations [28,33] utilized pre-trained knowledge, they remained bottlenecked by initial zero-shot errors. Our findings indicate that the EMA-based update rule allows the model to progressively refine its own targets, effectively acting as a self-correcting teacher. This is particularly evident in the results in the CoNSeP dataset, where our model significantly outperformed the baseline, suggesting that the self-evolving mechanism is crucial for capturing complex structural variations in colorectal adenocarcinoma tissues.

Another critical contribution is the implementation of the instance-aware contrastive learning module, which provides essential supervision in regions where pseudo-labels are ambiguous. A significant challenge in point-annotated nuclei segmentation is the instability of generated masks. In many cases, the initial prompt labels $S_{P}$ may fail to include any foreground regions due to high prediction entropy or disagreement between branches, leading to masks composed solely of background and ignored regions. Our contrastive learning approach addresses this by utilizing the coordinates of the point prompts as reliable spatial anchors. Even when the explicit binary supervision is missing or filtered out, the contrastive loss enforces feature consistency between the point-wise embedding and the instance prototype while maintaining discriminative distance from the background. This mechanism serves as a minimum learning guidance, ensuring that the model maintains a fundamental representation of the nuclei even in the absence of high-quality pseudo-masks. The ablation study confirms that this representational refinement consistently improves the AJI, which is particularly sensitive to missing or merged instances.

Furthermore, the hierarchical pseudo-label refinement strategy addresses the practical issue of training efficiency. In weakly supervised segmentation, the exclusion of ambiguous pixels often leads to a significant loss of training data. By leveraging the agreement between the mask decoder and the nuclei decoder, our framework recovers valid foreground and background pixels that would otherwise be ignored. This expanded data utilization not only improves final accuracy but also accelerates convergence, making the model more practical for nuclei segmentation, where computational resources may be limited.

## Figures and Tables

**Table 1:** Comparison results of nuclei instance segmentation on three public datasets. Shift 0 denotes precise point annotations at the nucleus center, while Shift 8 refers to noisy labels where the point prompt is randomly offset from the center by 0 to 8 pixels. Bold and underline indicate the best and second-best performance, respectively.

Method	CPM17				MoNuSeg				CoNSeP			
	Shift 0		Shift 8		Shift 0		Shift 8		Shift 0		Shift 8	
	Dice	AJI	Dice	AJI	Dice	AJI	Dice	AJI	Dice	AJI	Dice	AJI
MIDL [15]	75.0	55.5	72.2	49.9	70.1	44.9	66.9	41.8	–	–	–	–
Mixed Anno [16]	75.3	53.2	73.1	49.9	73.3	51.6	66.9	41.8	–	–	–	–
SPN + IEN [31]	74.3	54.3	69.4	46.8	74.0	53.4	65.6	39.4	–	–	–	–
PROnet [32]	78.7	62.7	77.0	60.2	75.0	55.5	72.5	50.9	62.1	41.4	58.3	35.8
All-in-SAM [33]	80.7	64.2	–	–	73.8	50.2	–	–	–	–	–	–
InstaSAM [25]	**83.9**	**69.5**	**82.4**	**67.2**	77.2	57.4	73.3	52.6	66.7	40.5	64.8	37.2
**Proposed**	82.9	68.0	82.0	65.6	**77.6**	**57.6**	**76.1**	**54.1**	**71.4**	**45.3**	**67.3**	**41.0**

**Table 2:** Comparison of different training strategies on CPM17 and MoNuSeg datasets using ViT-B backbone. Bold indicates the best performance.

Method	CPM17		MoNuSeg	
	Dice	AJI	Dice	AJI
InstaSAM [25] (Baseline)	79.20	63.49	73.85	51.39
EMA + Consensus Filtering	80.04	64.18	74.51	52.69
EMA + Consensus + Loss (Proposed)	**80.38**	**65.15**	**75.22**	**53.53**

**Table 3:** Ablation study of different EMA scheduling strategies ($\alpha_{t}$) on CPM17, MoNuSeg, and CoNSeP datasets using ViT-B backbone. Bold indicates the best performance.

Strategy	CPM17		MoNuSeg		CoNSeP	
	Dice	AJI	Dice	AJI	Dice	AJI
Constant (α=0.5)	80.88	65.60	73.85	51.39	56.32	28.55
Exponential (γ=0.5)	81.24	65.69	73.04	51.19	**62.19**	34.97
Step-wise (50/50 split)	**81.78**	**66.11**	73.92	52.27	59.82	32.68
Linear (Proposed)	80.38	65.15	**75.22**	**53.53**	61.04	**37.70**

**Table 4:** Backbone comparison on the CoNSeP dataset. Peak VRAM was measured during the training phase using a single NVIDIA RTX A6000. Bold indicates the best performance.

Backbone	Method	Dice	AJI	Peak VRAM (MB)
ViT-B	Baseline	54.44	27.68	77.12
	Proposed (Ours)	**61.04**	**37.70**	89.56
ViT-L	Baseline	61.67	34.30	137.98
	Proposed (Ours)	**67.60**	**41.92**	150.67
ViT-H	Baseline	66.70	40.48	184.62
	Proposed (Ours)	**71.44**	**45.30**	197.21

## Data Availability

The datasets utilized in this study—CPM17, MoNuSeg, and CoNSeP—are publicly available at their respective official repositories.
